# Circ_0003855 involvement of esophageal cancer progression through miR-622/FLOT1

**DOI:** 10.32604/or.2023.042447

**Published:** 2024-04-23

**Authors:** JINGJING TIAN, XIBAO HU, XINRONG ZHANG

**Affiliations:** 1 Department of Gastroenterology, Tianjin Nankai Hospital, Tianjin, 300100, China; 2 Department of Digestive Medicine, First Teaching Hospital of Tianjin University of Traditional Chinese Medicine, Tianjin, 300100, China

**Keywords:** Circ_0003855, miR-622, FLOT1, Esophageal cancer, Cell proliferation

## Abstract

To confirm the relationship between Circ_0003855 and EC, we purchased the Human esophageal carcinoma cell line Eca109 and normal human esophageal epithelial cells HEEC, and the expression levels of Circ_0003855, miR-622, and FLOT1 were detected. The results show that Circ_0003855 and FLOT1 were highly expressed in Eca109 cells, while miR-622 was lowly expressed (*p* < 0.05). Subsequently, Circ_0003855 small interfering RNA (si-Circ_0003855) and its negative control (si-NC) were used to detect changes in cellular biological behaviors. We found that the activity of Eca109 cells was reduced after interfering with the expression of Circ_0003855, and miR-622 expression was elevated, while FLOT1 was decreased (*p* < 0.05). Additionally, si-Circ_0003855 and miR-622 inhibitor sequence (miR-622-inhibition) were co-transfected into cells with miR-622-inhibition alone, and untreated Eca109 cells were used as a control to detect the expression of FLOT1. Co-transfection of si-Circ_0003855 and miR-622-inhibition showed no significant difference in FLOT1 expression compared to the control cells (*p* > 0.05). Synthesizing the results of these experiments above, we believe that interfering with the expression of Circ_0003855 can inhibit the activity of EC cells, and its mechanism is related to miR-622 and FLOT1.

## Introduction

Currently, the incidence of esophageal cancer (EC) is approximately 32/100,000, showing an increasing trend year by year in recent years [1]. On average, 150,000 people die from EC worldwide each year, and the 5-year prognosis survival rate is less than 20% [2]. At present, the pathogenesis of EC is not fully understood. Clinically, it is believed that multiple factors, such as poor dietary habits, long-term smoking and alcohol consumption, and viral infections, may contribute to the development of EC. In the early stages of the disease, there are usually no specific clinical symptoms and it may only present as a sensation of a foreign body when swallowing. However, by the time obvious pain occurs, the tumor has usually progressed to the middle or advanced stages [3,4]. Currently, there is still a lack of reliable and effective treatment options for advanced EC, which is one of the main reasons for poor prognosis in patients [5,6]. Therefore, finding new diagnostic and treatment strategies for EC has long been a hotspot and challenge in clinical research.

Circular RNA (circRNA) has been a hot research topic in the field of RNA in recent years [7,8]. CircRNA has a stable structure and does not degrade easily [9,10]. Several circRNAs have been shown to be associated with EC [11]. Among them, Circ_0003855 (CircDUSP16) is one of the newly discovered circRNA family members in recent years. It was initially considered as a candidate tumor suppressor in chromosomal region 12q13-13 [12]. As research progresses, it has been continuously confirmed to be closely related to gastrointestinal tumors such as colorectal and gastric cancers, and has been regarded as a breakthrough in the diagnosis and treatment of gastrointestinal tumors in the future [13,14]. However, we found that the research on Circ_0003855 in EC is relatively scarce. So far, only Ma et al. in 2021 proposed that Circ_0003855 is involved in the development of EC through the miR-497-5p/Transketolase Like Protein 1 (TKTL1) axis, but they also suggested that the mechanism of Circ_0003855 in EC may be related to other molecular substances [15].

It is well known that circRNAs can act as miRNA sponge molecules, further regulating downstream target proteins to exert their biological regulatory effects [16–18]. This has been validated in previous studies and in the research by Ma et al. [15]. Therefore, to further confirm the mechanism of Circ_0003855 on EC, we carried out related analyses on its downstream pathways. In the online target gene prediction website ENCORI, we preliminarily analyzed the downstream miRNAs of Circ_0003855, among which miR-622 caught our attention. As a miRNA closely related to tumors, miR-622 is considered an important tumor suppressor factor in EC, with significant potential effects [19,20]. Similarly, FLOT1, as one of the potential downstream marker proteins of miR-622, has been repeatedly confirmed to be associated with EC [21,22]. Thus, we hypothesize that the effect of Circ_0003855 on EC may be mediated through miR-622 and FLOT1.

Therefore, in order to further understand the relationship between Circ_0003855 and EC, this study will further confirm the effects of Circ_0003855 on the biological behavior of EC cells through *in vitro* experiments and explore the relationship with miR-622, FLOT1. This study will not only further validate the relationship between Circ_0003855 and EC, but will also confirm for the first time the mechanism of Circ_0003855’s effect on EC cells in addition to miR-497-5p/TKTL1. These findings will not only be beneficial to help clinics further understand the relationship between Circ_0003855 and EC, but also lay the foundation for future clinical development of Circ_0003855-based targeted therapies.

## Materials and Methods

### Cellular information

Human esophageal cancer cells Eca109 (BNCC342591) and human normal esophageal epithelial cells HEEC (BNCC359279) were purchased from Beina Biological Technology Co., Ltd. (Bejing, China), and cultured in a 90% DMEM high-glucose medium containing glutamine and sodium pyruvate, supplemented with 10% FBS, at 37°C and 5% CO_2_. Cell recovery: Cells were dissolved in a 37°C water bath, then added to a centrifuge tube containing 9mL complete medium, centrifuged for 5 min (1000 rpm/min), the supernatant was discarded, and the cells were resuspended in 1–2 mL complete medium. The cell suspension was added to a T25 bottle containing 5 mL of complete medium and incubated in an incubator. Cell passage: After the old culture medium was removed by suction and washed twice with PBS, 2 mL Trypsin (0.25% Trypsin + 0.02% EDTA) was added. The digestion was observed under a microscope, and the trypsin was removed by suction when the edge of the cells was reduced and the adherence was loosened. 5 mL of complete medium was added, and the cell layer was gently blown off and blown away. The cell suspension was divided at a 1:2 ratio into new T25 flasks, the above procedure was repeated when the cell growth density reached 70%–80%. The study protocol was approved by the Ethics Committee of Tianjin Nankai Hospital (Approval No. 2021-021).

### qPCR detection of Circ_0003855, miR-622, and FLOT1 expression

Total cellular RNA was extracted by Trizol (Thermo Fisher, Shanghai, China) and reversed to cDNA. GAPDH and β-actin used as internal references for amplification and PCR reactions of cDNA. 2^−ΔΔCT^ calculated quantitative results (Table 1).

**Table 1:** Primer sequences

	F (5′–3′)	R (5′–3′)
Circ_0003855	GCCCATGAGATGATTGGAACTC	CGGCTATCAATTAGCAGCACTTT
β-actin	GTGGCCGAGGACTTTGATTG	CCTGTAACAACGCATCTCATATT
miR-622	ATCCCAGGGAGACAGAGATCGAGG	AAGCTTGGTGGTGGACTTTTGGTTGT
U6	GGTGA AGCAGGCGTC GGAGG	GAGGGCAATGCCAGCCCCAG
FLOT1	GCAGAGAAGTCCCAACTAATTATGC	CAGTGTGATCTTATTGGCTGAAGTC
GAPDH	TGTTCGTCATGGGTGAAC	ATGGCATGGACTGGTCAT

### Western blot detection of FLOT1 expression

Protein purity was determined by bicinchoninic acid (BCA) assay (Sigma-Aldrich, Shanghai, China), and 20 μg of total protein was subjected to SDS-PAGE (Sigma-Aldrich, Shanghai, China) electrophoresis. The membrane was incubated overnight at 4°C with primary antibodies against FLOT1 and GAPDH (1:1000 dilution) (Abcam, Cambridge, UK). The next day, the membrane was washed to remove primary antibodies and incubated with secondary antibodies (1:5000 dilution) (Abcam, Cambridge, UK). Protein bands were visualized using enhanced chemiluminescent (Sigma-Aldrich, Shanghai, China) reagents, and the ImageJ software for analysis (National Institutes of Health, Washington, USA).

### Cell grouping and culture

Eca109 cells were transfected using lipid-mediated transfection and seeded in 96-well plates (3 × 10^3^ cells/well). Circ_0003855 small interfering RNA (si-Circ_0003855) and its negative control (si-NC) were diluted in serum-free medium and incubated for 5 min before being added to the 96-well plates according to the instructions of the Lipofectamine 2000 kit (Thermo Fisher, Shanghai, China). The transfected expression vectors were named si-Circ_0003855 group and si-NC group. Transfection efficiency was verified by detecting Circ_0003855 expression using qPCR. Additionally, cells co-transfected with si-Circ_0003855 and miR-622 inhibitor sequence (miR-622-inhibition), as well as cells transfected with miR-622-inhibition alone, were set up, with untreated Eca109 cells serving as control cells.

### Cell proliferation assay

Eca109 cells from each group were seeded in 96-well plates and incubated for 24, 48, 72, and 96 h. At each time point, 10 μL of CCK-8 (Sigma-Aldrich, Shanghai, China) solution was added to each well. The optical density (OD) values at 450 nm were measured using a microplate reader. Cells from each group were also seeded in 6-well plates (500 cells/well), when visible clones appeared in the 6-well plates, the culture was terminated, and the cells were fixed with 4% paraformaldehyde (Sigma-Aldrich, Shanghai, China) for 30 min, stained with 1% crystal violet for 30 min, and then air-dried. Photographs were taken.

### Cell invasion assay

After the Matrigel matrix solidified, 400 μL of antibiotic- and serum-free medium was added to the upper chamber, along with 200 μL of Eca109 cells (2 × 10^5^ cells/mL). The lower chamber was added with cell culture medium, and the cells were fixed with paraformaldehyde and stained (0.1% crystal violet, Sigma-Aldrich, Shanghai, China) after 24 h. The number of perforated cells was counted.

### Cell apoptosis assay

Transfected Eca109 cells were digested with trypsin, and resuspended. Then 100 μL cell suspension was placed in a clean centrifuge tube, and 5 μL AnnexinV-FITC and PI reagents were added in turn (E-CK-A211, Elabscience Biotechnology Co., Ltd., Wuhan, China).

### Statistical analysis

Results are expressed as means (±standard deviation). Statistical analysis was performed using SPSS 26.0 software. Group comparisons were conducted using *t*-tests, while comparisons among multiple groups or multiple time points were performed using analysis of variance (ANOVA) and LSD *post-hoc* tests. A *p* value of <0.05 was considered statistically significant.

## Results

### Circ_0003855 and FLOT1 were highly expressed in EC, and miR-622 was lowly expressed in EC

**Figure 1:**
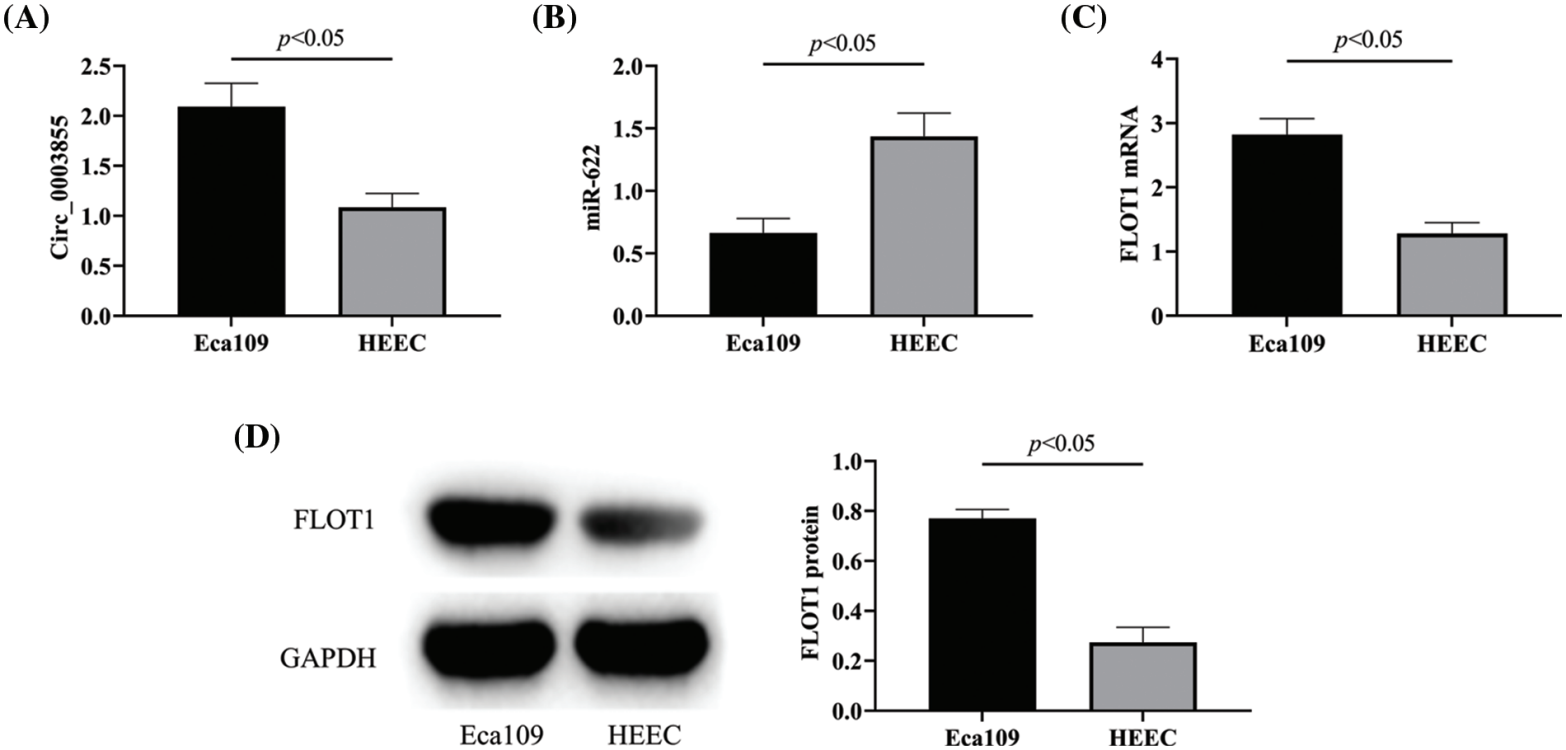
Circ_0003855, miR-622, and FLOT1 expression. (A) Comparison of Circ_0003855, (B) Comparison of miR-622, (C) Comparison of FLOT1 mRNA, (D) Comparison of FLOT1 protein. Circ_0003855 and FLOT1 in Eca109 are higher than HEEC, while miR-622 is lower than HEEC.

First, by comparing the expression levels of Circ_0003855, miR-622, and FLOT1 in Eca109 and HEEC cells, it was found that Circ_0003855 and FLOT1 mRNA expression were higher in Eca109 cells than in HEEC cells, while the expression of miR-622 was lower in Eca109 cells than in HEEC cells (*p* < 0.05, Figs. 1A–1C). Similarly, the Western blot results showed that FLOT1 protein expression in Eca109 cells was higher than in HEEC cells (*p* < 0.05), consistent with the qPCR detection results (Fig. 1D).

### Silencing Circ_0003855 expression can inhibit the growth of EC cells

**Figure 2:**
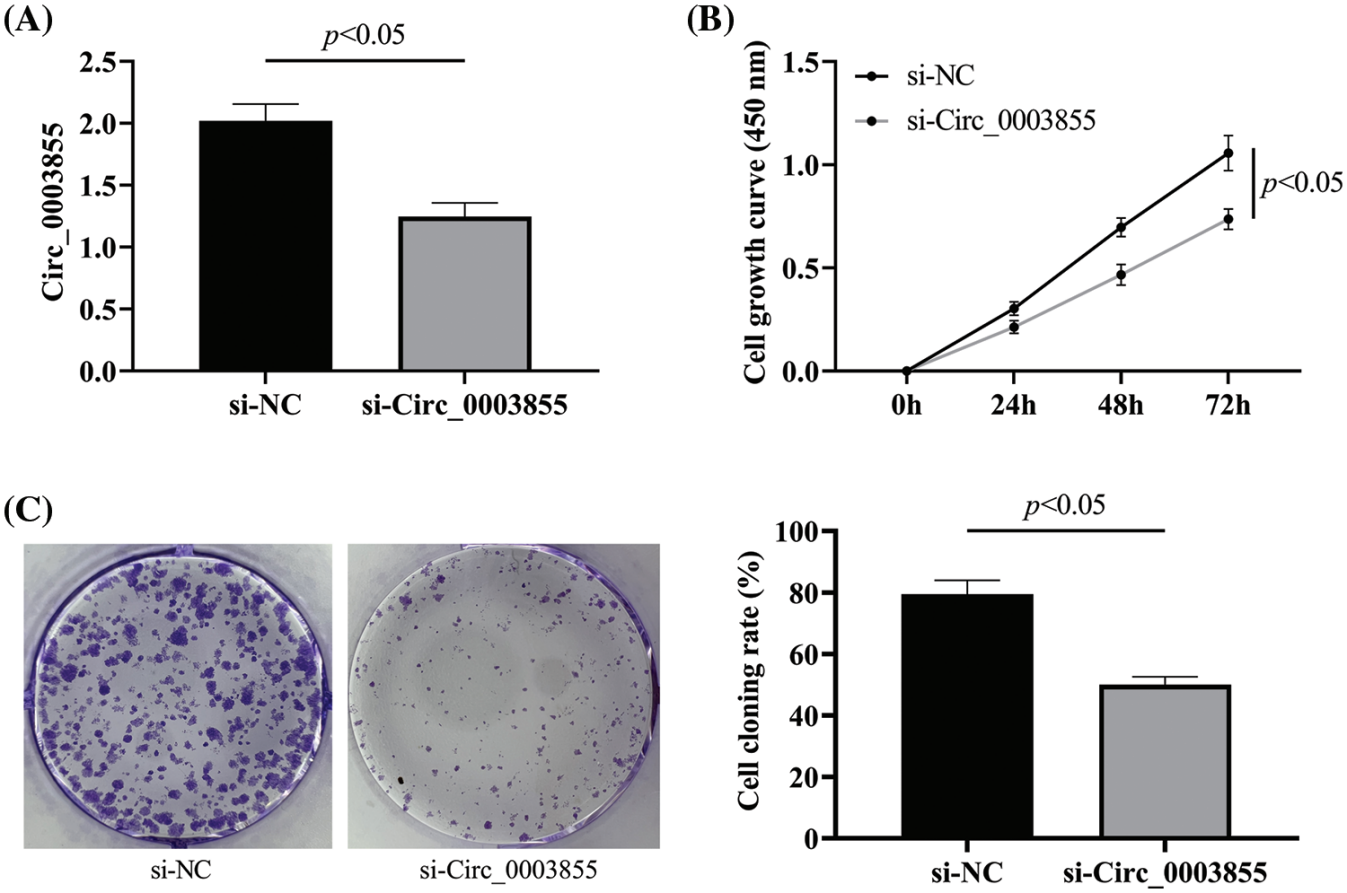
Effect of Circ_0003855 on the growth ability. (A) Expression of Circ_0003855. (B) Cell growth curve after transfection. (C) Results of colony formation experiments (50×). After silencing the expression of Circ_0003855 in Eca109, the growth ability of the cells was significantly weakened.

Detection of Circ_0003855 expression in Eca109 cells after transfection revealed that Circ_0003855 expression in the si-Circ_0003855 group was lower than in the si-NC group (*p* < 0.05, Fig. 2A), confirming successful transfection. In the CCK-8 assay, the cell growth curve of the si-Circ_0003855 group was significantly lower than that of the si-NC group (*p* < 0.05), and the clonal expansion situation in the si-Circ_0003855 group was also significantly reduced compared to the si-NC group (*p* < 0.05), indicating that interference with Circ_0003855 expression can inhibit the growth of Eca109 cells (Figs. 2B and 2C).

### Effects of Circ_0003855 on cellular invasion

**Figure 3:**
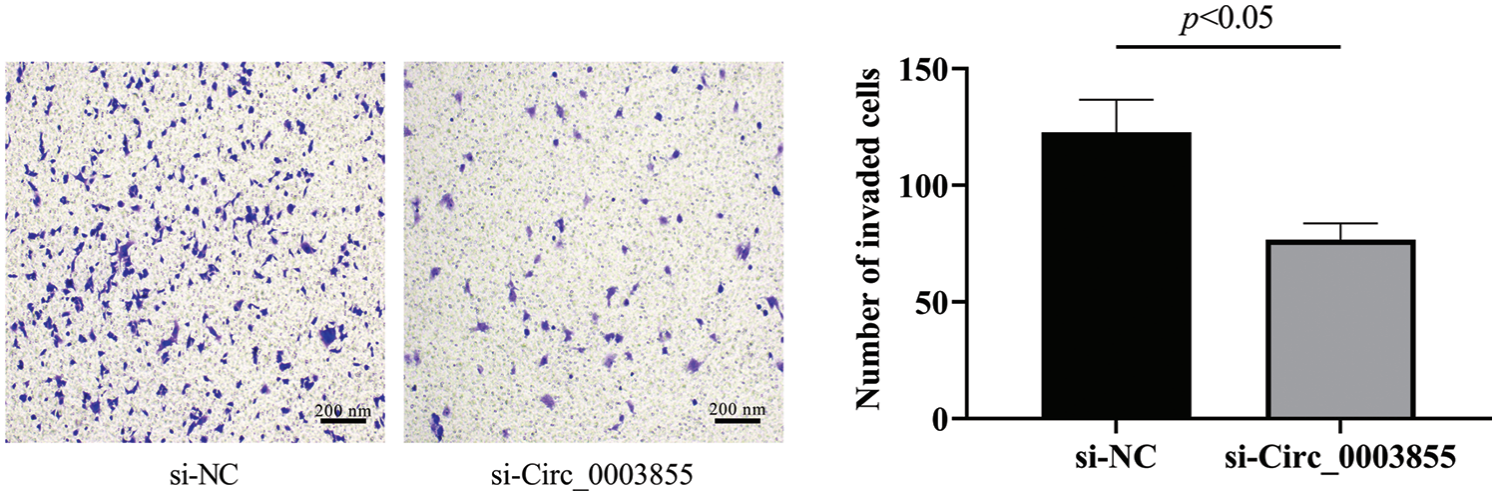
Transwell assay to detect the effect of Circ_0003855 on the invasive ability of Eca109 (100×). After silencing the expression of Circ_0003855 in Eca109, the invasion ability of cells was significantly weakened.

Subsequently, in the Transwell assay, it was found that the number of cells penetrating the membrane in the si-Circ_0003855 group was about (76.67 ± 7.02), which was significantly lower than that in the si-NC group (*p* < 0.05), indicating that interference with Circ_0003855 expression can inhibit the invasion ability of Eca109 cells (Fig. 3).

### Silencing the expression of Circ_0003855 can promote the apoptosis of EC cells

**Figure 4:**
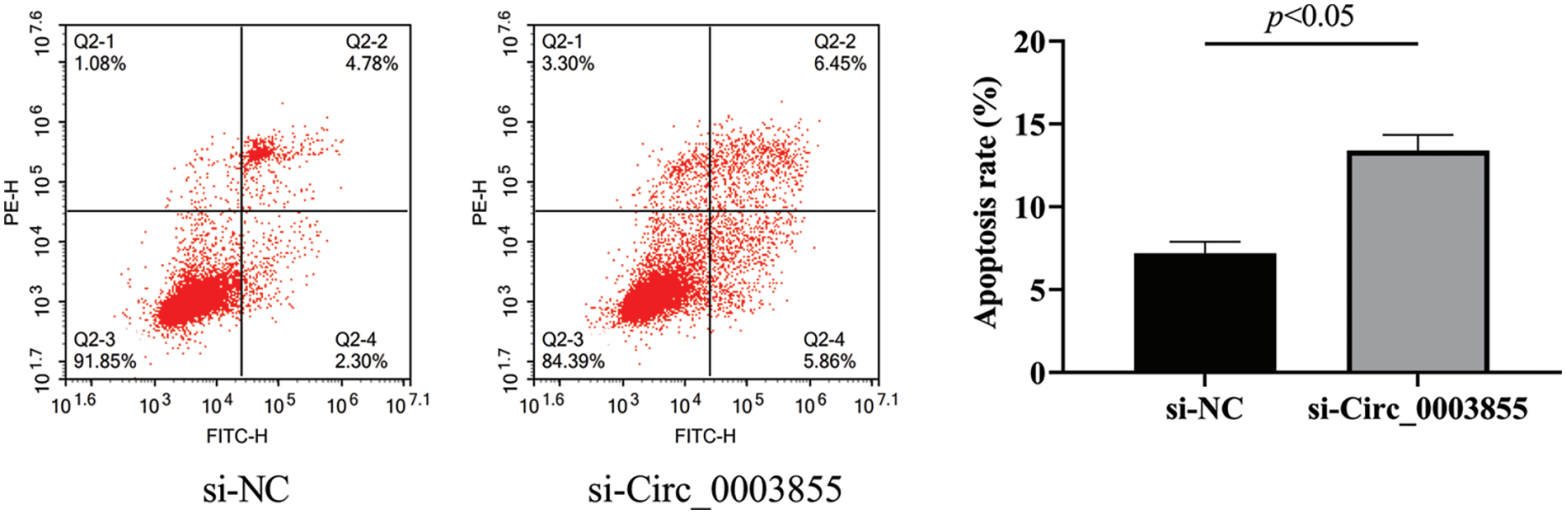
Flow cytometric assay of Circ_0003855 on the apoptosis rate of Eca109. After silencing the expression of Circ_0003855 in Eca109, the apoptosis of cells was significantly enhanced.

Flow cytometry results showed that the apoptosis rate of cells in the si-Circ_0003855 group was (13.39% ± 0.95%), while the apoptosis rate in the si-NC group was (7.19% ± 0.71%). indicating that the apoptosis rate in the si-Circ_0003855 group was higher (*p* < 0.05). Overall, this sugges that the interference with Circ_0003855 expression can promote apoptosis in Eca109 cells (Fig. 4).

### Circ_0003855 can inhibit miR-622 and increase the expression of FLOT1 in EC cells

**Figure 5:**
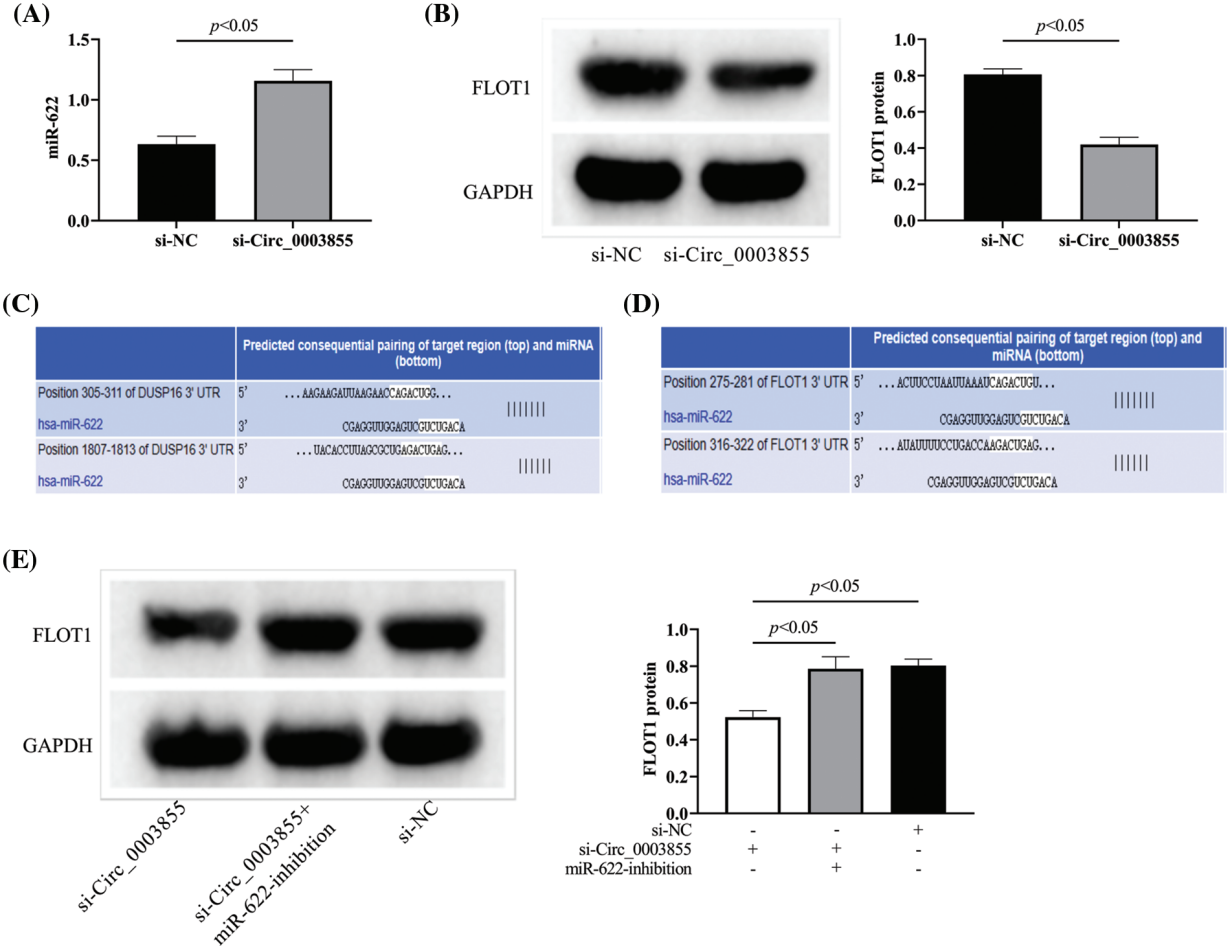
Effect of Circ_0003855 on the expression of miR-622 and FLOT1. (A) Effect of Circ_0003855 on miR-622 expression level. (B) Effect of Circ_0003855 on FLOT1 protein expression. (C) Bindable complementary sites for Circ_0003855 and miR-622. (D) Bindable complementary sites for miR-622 and FLOT1. (E) Effect of simultaneous inhibition of Circ_0003855 and miR-622 on FLOT1 protein expression. After silencing the expression of Circ_0003855 in Eca109, the expression of miR-622 was increased, while the expression of FLOT1 was decreased. After silencing the expression of Circ_0003855 and miR-622 in Eca109 at the same time, the expression of FLOT1 was not different from that of si-NC group.

By detecting the expression of miR-622 and FLOT1 in the si-Circ_0003855 and si-NC groups, it was found that miR-622 was higher in the si-Circ_0003855 group than in the si-NC group, while FLOT1 was lower than in the control group (*p* < 0.05, Figs. 5A and 5B). The bindable complementary sites between Circ_0003855, miR-622 and FLOT1 are shown in Figs. 5C and 5D (TargetScan, https://www.targetscan.org/vert_72/). Finally, by co-transfecting si-Circ_0003855 and miR-622-inhibition, we found that the expression of FLOT1 in Eca109 cells showed no significant difference compared to the control cells (*p* > 0.05), all higher than si-Circ_0003855 group (*p* < 0.05), suggesting that Circ_0003855 and miR-622 may have a role in targeting and regulating FLOT1 (Fig. 5E).

## Discussion

There is still a great potential threat to EC [23–25]. Although many studies have demonstrated the relationship between Circ_0003855 and gastrointestinal tumors, the specific mechanism in EC still needs further investigation. Therefore, we conducted a more in-depth analysis.

First, we found that Circ_0003855 was significantly higher in Eca109 cells than in HEEC, indicating that Circ_0003855 is overexpressed in EC. Previous studies on Circ_0003855 in malignant tumors have also shown that Circ_0003855 is elevated in tumor tissues (cells) [12,26], which is consistent with our experimental results. In previous studies, we found that Circ_0003855 can participate in osteosarcoma progression through miR-145-5 [14,27], which fully demonstrates the importance of Circ_0003855 in tumor development. Moreover, Ma et al. believed that Circ_0003855 inhibits hypoxia-induced EC cell growth, invasion, and glycolysis by sponging miR-497-5p to regulate TKTL1 expression [15]. However, the limitation of their study is that they did not analyze the effect of Circ_0003855 alone on EC cells. Based on this, the results showed reduced activity and enhanced apoptosis in the si-Circ_0003855 group. The results showed that the cell growth and invasion abilities in the si-Circ_0003855 group were significantly inhibited, and the apoptosis rate increased. This supports the experimental results of Ma et al., indicating that overexpressed Circ_0003855 plays an oncogene role in EC, and silencing its expression can inhibit the progression of EC. Similarly, the study by Li et al. also showed that after downregulating Circ_0003855 expression, the activity and migration ability of liver cancer cells were inhibited [28]. The above experimental results also suggest that Circ_0003855 may be a new therapeutic target for EC, which undoubtedly has important clinical significance and social benefits for patients with mid-to-late-stage EC, who currently lack effective treatment options.

Subsequently, we found miR-622 was underexpressed in EC cells, while FLOT1 was overexpressed. Consistent with previous studies. References [29,30] supported our experimental results. In addition, a recent study showed that miR-622 plays a role as a tumor suppressor in EC [20], which also fully confirmed the important significance of miR-622 in EC. At the same time, Wu et al. also mentioned in the study that the migration and invasion ability of EC cells was enhanced after the expression of miR-622 was increased [31], which was also consistent with the down-regulated state of miR-622 in Eca109 in this study. As for FLOT1, the study of Song et al. also confirmed its promoting effect on EC [21], and Zhao et al. found that after inhibiting the expression of FLOT1, the epithelial-mesenchymal transition (EMT) of EC cells can be improved and the growth of EC can be reduced [22], which is also consistent with our view. Moreover, after interfering with Circ_0003855 expression, we observed an increase in miR-622 expression and a decrease in FLOT1. At the same time, when downregulating Circ_0003855 and miR-622, the expression of FLOT1 in Eca109 cells was the same as in controls. This suggests that Circ_0003855 and miR-622 have a target competitive binding relationship and regulate the expression of FLOT1, thereby affecting the biological behavior of EC cells. Of course, the relationship between the three still needs further research for confirmation.

Due to experimental conditions, there are still many shortcomings in this study. As mentioned above, we still need more experiments to confirm the effects of Circ_0003855, miR-622, and FLOT1 on EC and the relationship among them (such as dual luciferase reporter, immunoprecipitation, etc.), as well as to further confirm the changes in tumor growth *in vivo* after interfering with Circ_0003855 expression. In addition, we also need to conduct clinical trials as soon as possible to observe the expression of these factors in actual EC cases. In the future, we will conduct more in-depth research and analysis on these limitations to provide more comprehensive reference and guidance for clinical practice.

## Conclusion

Circ_0003855 is highly expressed in EC, and its interference can inhibit the activity of EC cells. In the future, it is expected to become a new direction for EC diagnosis and treatment research. The mechanism may be mediated through miR-622 and FLOT1, which still requires more experiments for confirmation.

## Data Availability

The data in this article can be obtained from the corresponding author under reasonable circumstances.
